# Beneficial Effects on Pregnancy Outcomes of Thyroid Hormone Replacement for Subclinical Hypothyroidism

**DOI:** 10.1155/2017/4601365

**Published:** 2017-02-14

**Authors:** Norman J. Blumenthal, Creswell J. Eastman

**Affiliations:** ^1^Notre Dame University, Sydney, NSW, Australia; ^2^Sydney Medical School, University of Sydney, Sydney, NSW, Australia

## Abstract

*Background*. Hypothyroidism and raised thyroid antibody levels have been associated with adverse obstetrical outcomes. Several studies have investigated causal associations, but results have been inconsistent and few studies have reported the effects of thyroxine replacement therapy on pregnancy outcomes in hypothyroid patients.* Objective*. The primary study objective was to determine the outcome of pregnancies in women diagnosed with overt and subclinical hypothyroidism (SCH) (serum TSH > 2.5 mIU/L) and those with elevated circulating thyroid autoantibody levels in the first trimester of pregnancy and after the institution of appropriate thyroxine replacement therapy to maintain the serum TSH ≤ 2.5 mIU/L.* Study Design*. This prospective observational study was undertaken between 2013 and 2016. Blood samples were taken from 1025 women at presentation for thyroid stimulating hormone (TSH), anti-thyroglobulin antibodies (TGAb), and thyroid peroxidase antibodies (TPOAb). Those with a TSH > 2.5 mIU/L were treated with thyroxine and managed appropriately to ensure that the TSH was maintained ≤2.5 mIU/L. Outcomes in these patients were compared to those in euthyroid patients. Maternal antenatal complications and perinatal outcomes were recorded.* Results*. There were a total of 1025 patients of whom 382 (37.5%) were nulliparous. 10.1% had a TSH level > 2.5 mIU/L and 18.2% had at least one raised thyroid antibody level. No differences in adverse outcomes of pregnancy were evident in women treated for SCH or overt hypothyroidism compared to the euthyroid group. There was also no association between raised thyroid antibodies and adverse pregnancy outcomes in either group.* Conclusion*. There were no adverse outcomes of pregnancy found in pregnant women who had been diagnosed and treated with thyroxine for SCH at the time of presentation when compared to euthyroid patients. There was also no relationship with thyroid antibodies and adverse pregnancy outcomes in the two groups. It is not possible to unequivocally advocate for thyroxine replacement in pregnant women with subclinical and overt hypothyroidism until large scale randomized controlled trials are performed.

## 1. Introduction

Thyroid disorders are among the most common endocrine disorders in pregnancy. The prevalence of hypothyroidism in pregnancy varies from 0.4% to 11% worldwide [1]. The definitions of subclinical and overt hypothyroidism vary and this may account for some of the different reports of prevalence. In addition, each laboratory should have its own pregnancy-specific reference ranges for thyroid function tests. Current American Endocrine Society (AES) Guidelines define overt hypothyroidism during the first trimester of pregnancy as a patient with a serum TSH level > 10 mIU/L with or without a subnormal serum free thyroxine (FT4) level and subclinical hypothyroidism (SCH) as a patient with a serum TSH level > 2.5 mIU/L with a normal free T4 level [2]. The American Thyroid Association (ATA) defines SCH as a TSH between 2.5 and 10 mIU/L with a normal FT4 concentration [1].

Early detection is important because SCH has been associated with numerous adverse effects on the outcome of pregnancy such as increased rates of preeclampsia, gestational diabetes, preterm birth, induction of labour, and caesarean section [3, 4].

Autoimmune thyroid disease (AITD), characterized by raised antibodies to either thyroperoxidase (TPOAb) or thyroglobulin (TGAb), also has a reasonably high prevalence during pregnancy and varies widely from 5 to 15% [3]. Elevated circulating levels of thyroid antibodies may not necessarily be associated with thyroid dysfunction but, in their own right, have been associated with an increase in various adverse outcomes of pregnancy and have been suggested as being independent markers for at-risk pregnancies [5]. The mechanism for the adverse outcomes and at what concentration this occurs are not fully understood.

We therefore sought to determine possible beneficial effects of thyroxine treatment for overt and subclinical hypothyroidism on adverse maternal and fetal outcomes in pregnancy compared with an untreated euthyroid control group and also determine possible independent adverse effects in those women who were thyroid autoantibody positive.

*Objective*. The primary objective of the study, undertaken in a private obstetrical practice in Sydney, was to determine the outcomes of those pregnancies in patients who had been treated with thyroxine for subclinical and overt hypothyroidism and compare them to the outcomes in untreated patients with normal TSH levels. The secondary objective was to assess the effect of thyroid autoimmunity in both groups.

## 2. Materials and Methods

This study was performed in a private practice setting in North Western Sydney between 2013 and 2016 where 1025 new consecutive antenatal patients were reviewed in their first trimester and relevant information about these patients results of investigations was provided in the initial published report [6]. This population group is from middle to upper socioeconomic group and all were nonsmokers. None were taking any form of thyroid hormone replacement therapy. Data was not recorded on ethnicity and a previous history of small for gestational age (SGA) babies. At the time of initial booking with the clinic, between 7 and 11 weeks of gestation, the routine antenatal investigations were performed. At the same time, assessment of thyroid function was performed by measurement of TSH and FT4 levels, TGAb and TPOAb. Patients were considered to have SCH if the TSH was >2.5 mIU/L but <10.0 mIU/L with a normal FT4 level and overt hypothyroidism if the TSH was >10.0 mIU/L regardless of the FT4 level. Few patients with a TSH < 2.5 mIU/L and a subnormal FT4 level were classified as “isolated hypothyroxinemia” and were not treated as hypothyroid. Mothers were considered to be antibody positive if either the TGAb or the TPOAb were >60 IU/mL. All those with a raised TSH (>2.5 mIU/L) were initially treated with thyroxine 50 ug daily to keep the TSH level ≤ 2.5 mIU/L, as recommended by the American Endocrine Society Guidelines (1). Thyroxine replacement was continued throughout pregnancy, with TSH monitoring every 4 weeks in the first and second trimester and once in the third trimester. Adjustment of dosage was made appropriately to maintain serum TSH levels between 0.1 mIU/L and 2.5 mIU/L. Patients with a TSH of ≤2.5 mIU/L, regardless of FT4 level, were considered euthyroid and were not treated. A glucose challenge test was performed between 26 and 28 weeks of gestation on all women using a one-hour nonfasting sugar level after a 50 gm load, as suggested at that time by the Australian Diabetes in Pregnancy Association (ADIPS). A full glucose tolerance test was only performed if the challenge glucose response was ≥7.8 mmol/L. The medical records of each patient were then reviewed at the end of the pregnancy. Maternal antenatal conditions such as pregnancy induced hypertension, antepartum haemorrhage, and abruption of the placenta were recorded. Documentation of fetal outcomes of each pregnancy included information on premature labour (birth < 37 completed weeks of gestation), premature rupture of the membranes, SGA (birth weight less than 10th centile according to the World Health Organization (WHO) customized fetal growth charts), stillbirth, fetal death in utero, and caesarean section.

Informed consent was obtained from each woman who participated in this study. Women with multiple pregnancies were excluded from the study. All women were routinely prescribed iodine supplementation from the time of first presentation to eliminate any possible adverse effects of iodine deficiency on the pregnancy.

We were unable to have an untreated control group, as this study would not have gained ethics approval. Our hospital guidelines follow those of the AES which recommend treatment for all pregnant women, thyroid antibody positive or not, with thyroxine [2]. We do note that the ATA recommends treatment for only those patients who have a raised TSH and positive thyroid antibodies [1]. Other authoritative sources also recommend universal treatment of patients with SCH in pregnancy [1]. Therefore, our control group was the untreated euthyroid pregnant patients.

The Ethics Committee of the Sydney West Area Health Service and the Human Research Ethics Committee of The University of Notre Dame have approved the study.

### 2.1. Serum Thyroid Stimulating Hormone (TSH) and Thyroid Antibody Measurements

Serum TSH levels were measured by a chemiluminescent immunoassay on the ADVIA Centaur platform (Bayer Health Care) by Laverty Pathology laboratories in Sydney. The details of the performance of these assays have been previously reported [6].

Quantitative thyroid antibodies were measured using the ADVIA Centaur XP System. Thyroperoxidase antibodies (TPOAb) and anti-thyroglobulin (TGAb) antibodies were considered positive above a level of 60 international units (IU)/mL as recommended by the manufacturer (Siemens). All laboratory determinations were carried out in the same laboratory. The results of serum TSH and thyroid antibody testing have previously been reported [6].

### 2.2. Statistical Analysis

The statistical software package IBM SPSS v22 was used to analyse the data. Two-tailed tests with a significance level of 5% were used throughout. Chi-squared or Fisher's exact tests, as appropriate, were used to test for association between categorical variables. Odds ratios and their 95% confidence intervals (95% CI) were used to quantify the extent of association with dichotomous outcomes. The Mann–Whitney test or Kruskal-Wallis nonparametric analysis of variance was used to test for differences in the distribution of continuous variables by group. Spearman's rank correlation (*r*) was used to quantify the degree of association between continuous and ordered categorical variables.

## 3. Results

There was a total of 1025 patients of whom 382 (37.5%) were nulliparous. 10.1% had a TSH level > 2.5 mIU/L. Eighty-seven women (8.5%) had TSH levels > 2.5 and ≤5 mIU/L, while twelve women (1.2%) had a TSH > 5 and ≤10 mIU/L. There were 4 patients (0.4%) with a TSH greater than 10 mIU/L. 18.2% had raised TPOAb and 13.6% had raised TGAb levels. Both antibodies were detected in 88 patients (8.6%) (95% CI 7.0% to 10.5%), and 18.2% had at least one raised thyroid antibody.

The mean maternal age in the untreated group was 35 years (SD 5.2) and that of the treated group 33.47 years (SD 1.41). There was a statistically significant difference between the age groups (*p* = 0.002); but, while this is statistically significant, a difference of 2 years is not clinically important in these patients. The mean body weight was 70.16 kg (SD 18.96) in the untreated group and 70.01 kg (SD 0.71) in the treated patients, with no significant difference existing between these 2 groups (*p* = 0.28). A normal vaginal delivery occurred in 574 patients (56%) and with forceps or vacuum extraction in 120 patients (11.7%). There was no significant difference in caesarean section rate of 30.02% in the untreated group and 35.6% in the treated group (*p* = 0.41).

Gestational diabetes was recorded in 39 patients (3.8%). The prevalence of pregnancy induced hypertension (hypertension developing after 20 weeks of gestation) was 2% (20 patients). Premature labour occurred in 19 patients (1.9%), antepartum haemorrhage in 5 (0.5%), abruption of the placenta in 1 patient (0.1%), premature rupture of the membranes in 20 (2.0%), and SGA in 18 (1.8%) of patients. A stillbirth or fetal death in utero (>20 weeks of gestation) occurred in 14 patients (1.4%) (Table 1).

There was no statistically significant difference between the group of thyroxine treated hypothyroid patients and untreated euthyroid women in documented complications of pregnancy, such as pregnancy induced hypertension, antepartum haemorrhage, abruption of the placenta, premature labour, and premature rupture of membranes. However, prevalence of SGA occurred in 4.3% of the 92 pregnancies in the treated group and was close to significance (*p* = 0.050) (4.3% versus 1.5%), (Table 1).

The outcomes of pregnancy in those patients with any raised antibody are shown in Table 2 with no statistically significant associations with adverse outcomes of pregnancy.  Table 3 shows the outcomes of pregnancy in the group of women who had at least one raised antibody (TPOAb or TGAb). In this group, none of the parameters reached any statistical significance, not even those with SGA (*p* = 0.443).

The numbers were statistically too small to do a subanalysis on those patients with clinical and chemical hypothyroidism.

## 4. Comment

We have recently reported a high rate of SCH and raised thyroid antibodies in this cohort of patients [6]. We subsequently followed this cohort of patients through to delivery, treating those with overt or SCH with thyroxine, to document possible adverse obstetric and perinatal outcomes and to ascertain if there was a beneficial effect of thyroxine treatment in preventing adverse outcomes. While a number of studies have followed pregnant patients suffering from SCH to determine adverse obstetrical and fetal outcomes, at the time we conducted this study, there was little or no data examining the possible beneficial effects of thyroxine treatment in preventing adverse outcomes.

Reference ranges for TSH during the first trimester of pregnancy remain controversial and there may be geographical and ethnic factors to take into account. We have used the reference ranges recommended by the American Endocrine Society Guidelines with the upper limit of the euthyroid pregnant reference range in the first trimester being 2.5 mIU/L.

In a recent Cochrane review, no studies were identified that assessed the outcome of treatment with thyroxine on pregnant women with SCH [7]. In our study, patients with SCH and overt hypothyroidism were treated with thyroxine from the first antenatal visit to maintain normal thyroid function (serum TSH 0.1–2.5 mIU/L) and we then assessed the outcomes of pregnancy. This study was therefore different to most previously reported studies that looked for an association or cause and effect relationship between SCH diagnosed in the first trimester and adverse outcomes of the pregnancy. Our affected patients were treated with thyroxine and there was no control group of untreated hypothyroid patients, as our hospital current guidelines preclude nontreatment of patients with SCH and this is also recommended by AES. However, our control group was the untreated euthyroid patients, who were a similar population group. Our patients were monitored by regular TSH testing and adjustment of thyroxine dosage to ensure that the serum TSH levels were maintained between 0.1 mIU/L and 2.5 mIU/L.

We found no difference in outcome between our 2 groups. However, an interesting outcome finding in this study was that women with SCH (92 patients), even after correction of thyroid function with thyroxine replacement therapy, had an increased rate of SGA, 4.3% compared with 1.5% in the euthyroid control group, but this was not statistically significant (*p* = 0.05) and the number of affected babies was only 4. In a recently published study, Maraka et al. from the Mayo Clinic reported a retrospective study where they had a treated and an untreated hypothyroid control group and showed that SGA was significantly decreased in the thyroxine treatment group (1.3% versus 10%; *p* < 0.001) [8].

The prevalence of SGA in SCH women has been well documented in several reviews [9, 10] and in other studies [11, 12] but in none of these studies has the SCH been treated at initial presentation. In spite of the fact that we treated SCH patients with thyroxine at presentation and maintained the serum TSH at <2.5 mIU/L throughout pregnancy, the rate of SGA was 4.3%, raising the possibility that other factors or mechanisms may contribute to this adverse outcome. However, this conclusion needs to be considered with caution, as the number of women delivering babies with SGA in our study was very small (*n* = 4). If there is a direct cause and effect relationship between women suffering SCH and subsequently delivering a baby with SGA, one of the factors may be the timing of testing and of commencement of appropriate thyroxine therapy. Our patients presented between 7 and 11 weeks of gestation so it is possible that this delay in initiating thyroxine replacement therapy could be a contributory factor. We also accept that even though our patients may be a relatively homogenous group, there may have been a difference in characteristics between the 2 groups.

The mean maternal age in the present study was 34.9 years and this is higher than most of the other reports. This may partly account for our higher prevalence of SCH and AITD as both SCH and AITD have been shown to increase with age [13]. We reported no increase in pregnancy complications in relation to parity and Walsh and colleagues reported similar findings [14].

No other significant adverse outcomes of pregnancy in relation to treated SCH were documented in this study. This is in contrast to other studies [15, 16] of untreated patients that have shown an increase in adverse outcomes. Of interest, is that Schneur and colleagues, also found that high TSH levels in the first trimester in 2801 women were associated with adverse pregnancy outcomes especially SGA. However, the predictive accuracy was reported to be poor [17]. By contrast, Ong and colleagues, although documenting a 15% adverse outcome rate, suggested that testing for a raised TSH in the first trimester does not predict adverse outcomes of pregnancy [18].

Although increased rates of SGA have been shown to be present in women with clinical hypothyroidism or SCH, there have been few studies where an association between AITD and SGA has been documented [11, 19]. Negro and colleagues found that, in euthyroid pregnant women, who were positive for TPOAb and received thyroxine treatment, there was a lowering of the chances of miscarriage and premature delivery [20] and our study supports the lower prevalence of adverse pregnancy outcomes on thyroxine replacement.

We have shown no increase in other adverse outcomes of pregnancy associated with the presence of increased thyroid antibodies except for a correlation with a raised TSH (Table 2). However, a possible association may be confounded by the fact that patients with SCH and thyroid antibodies were treated with thyroxine. In other studies, an increase in the risk of placental abruption, premature labour, gestational diabetes, and severe preeclampsia has been shown with raised antibodies [21–25]. The prevalence of gestational diabetes in this study is lower than reported elsewhere and this may be due to the motivated, generally nonobese, and higher socioeconomic status of patients in the study.

In a very recent publication and in a large study, Plowden has shown that a high TSH level is not associated with infertility, pregnancy loss, or a decrease in live birth rate. This is different to our study as we mainly looked at the other adverse outcomes of pregnancy [26].

The strengths of our study are that one person in a single practice provided the obstetric care to all of the patients who were generally from a higher socioeconomic group. Compared to reported poor outcomes of untreated patients with SCH, we have shown no adverse outcomes after treatment.

The weaknesses of the study are that we do not have a matched control group for the reasons provided. However, we did compare our treated group with a large unselected group of euthyroid pregnant women. The other weakness is the small sample size. The present study was conducted more as an audit to get estimates of rates of the different outcomes for women with normal thyroid function and those treated for SCH. As SGA is the only outcome with an appreciable difference, the power calculation was done with respect to only SGA. To detect an increase of 2.8% in the rate of SGA from 1.5% in women without SCH to 4.3% in women with SCH, with 80% power and a significance level of 5%, 3500 women would be required, assuming the proportion of women with SCH is approximately 9% (310 women with SCH and 3190 without).

Our study supports the view that thyroxine replacement in pregnant women suffering from overt or SCH does prevent obstetric and fetal complications and shows no harmful effects of thyroxine therapy. However, without a matched untreated control group, we cannot conclude that all pregnant women with SCH should be treated with thyroxine. Negro and Stagnaro-Green have suggested in a preliminary intervention trial that treatment of SCH in early pregnancy is beneficial and is to be recommended [27]. If this is to become accepted best practice, it follows that universal screening for an elevated TSH will need to be implemented as early as possible after confirmation of conception and patients will need to be treated and monitored throughout their pregnancies.

## Figures and Tables

**Table 1:** A comparison of pregnancy outcomes in patients with initial serum TSH ≤ 2.5 mIU/L (*n* = 933) and those with serum TSH > 2.5 mIU/L who were treated with thyroxine (*n* = 92).

Variable	Initial TSH ≤ 2.5 mIU/L		Initial TSH > 2.5 mIU/L		*p* value	Odds ratio	LC	UC
	(*N* = 933)		(*N* = 92)					
	*n*	%	*n*	%				
TGAb > 60 IU/mL	111	11.9	28	30.1	0.001	3.2	2.0	5.2
TPOAb > 60 IU/mL	141	15.1	46	49.5	0.001	5.5	3.5	8.6
GTT positive (GD)	36	3.9	3	3.2	0.760	0.8	0.3	2.8
Nulliparous	346	37.2	37	40.2	0.574	1.1	0.7	1.8
APGAR 1 minute < 7	32	3.5	1	1.1	0.220	0.3	0.1	2.3
APGAR 5 minutes < 7	7	0.8	0	0.0	0.402	0.9	0.9	0.9
Fetal death in utero (<26 weeks)	5	0.5	0	0.0	0.479	0.9	0.9	0.9
Stillbirth or FDIU	13	1.4	1	1.1	0.800	0.8	0.1	5.9
Pregnancy induced hypertension	18	1.9	2	2.2	0.884	1.1	0.3	4.9
Premature labour	16	1.7	3	3.2	0.304	1.9	0.6	6.7
Antepartum haemorrhage	5	0.5	0	0.0	0.479	0.9	0.9	0.9
Abruption	1	0.1	0	0.0	0.752	0.9	0.9	0.9
SROM	19	2.0	1	1.1	0.522	0.5	0.7	4.0
SGA	14	1.5	4	4.3	0.050	2.9	1.0	9.1

TSH, thyroid stimulating hormone; TGAb, anti-thyroglobulin antibodies; TPOAb, thyroperoxidase antibodies; SROM, spontaneous rupture of membranes; SGA, small for gestational age; FDIU, fetal death in utero.

**Table 2:** A comparison of pregnancy outcomes in women with and without detectable circulating thyroid antibodies.

Variable	No positive antibody		≥1 positive antibody		*p* value	Odds ratio	LC	UC
	(*N* = 789)		(*N* = 236)					
	*n*	%	*n*	%				
TGAB > 60 IU/mL	0	0.0	139	58.4	N/A	0.1	0.1	0.1
TPOAb > 60 IU/mL	0	0.0	187	78.6	N/A	0.1	0.1	0.1
TSH > 2.5	46	5.8	47	19.7	0.0001	4.0	2.6	6.2
GTT positive (GD)	28	3.6	10	4.3	0.638	1.2	0.6	2.5
Nulliparous	301	38.6	79	34.2	0.226	0.8	0.6	1.1
APGAR 1 minute < 7	25	3.2	8	3.5	0.861	1.1	0.5	2.4
APGAR 5 minutes < 7	5	0.6	2	0.9	0.724	1.3	0.3	7.0
Fetal death in utero (<26 weeks)	3	0.4	2	0.9	0.371	2.2	0.4	13.4
Stillbirth or FDIU	10	1.3	4	1.7	0.628	1.3	0.4	4.3
Pregnancy induced hypertension	17	2.2	3	1.3	0.383	0.6	0.2	2.0
Premature labour	15	1.9	4	1.7	0.827	0.9	0.3	2.7
Antepartum haemorrhage	3	0.4	2	0.9	0.371	2.2	0.4	13.4
Abruption	1	0.1	0	0.0	0.583	0.8	0.7	0.9
SROM	19	2.4	1	0.4	0.052	0.2	0.0	1.3
SGA	11	1.4	7	3.0	0.110	2.1	0.8	5.6

TSH, thyroid stimulating hormone; TGAb, anti-thyroglobulin antibodies; TPOAb, thyroperoxidase antibodies; SROM, spontaneous rupture of membranes; SGA. small for gestational age; FDIU, fetal death in utero.

**Table 3:** Pregnancy outcome with any raised antibody and with TSH < 2.5 mIU/L.

Variable	No positive antibody		≥1 positive antibody		*p* value	Odds ratio	LC	UC
	(*N* = 742)		(*N* = 191)					
	*n*	%	*n*	%				
TGAb > 60 IU/mL	0	0.0%	111	58.1	NA			
TPOAb > 60 IU/mL	0	0.0%	141	73.8	NA			
GTT positive (GD)	28	3.8%	7	3.7	0.956	1.0	0.4	2.3
Nulliparous	281	38.2%	62	33.5	0.236	0.8	0.6	1.1
APGAR 1 minute < 7	24	3.3%	8	4.3	0.495	1.3	0.6	3.0
APGAR 5 minutes < 7	5	0.7%	2	1.1	0.582	1.6	0.3	8.2
Fetal death in utero (FDIU)	3	0.4%	2	1.1	0.274	2.6	0.4	15.8
Stillbirth or FDIU	10	1.4%	3	1.6	0.807	1.2	0.3	4.3
Pregnancy induced hypertension	15	2.0%	3	1.6	0.694	0.8	0.2	2.7
Premature labour	13	1.8%	3	1.6	0.817	0.9	0.3	3.2
Antepartum haemorrhage	3	0.4%	2	1.1	0.274	2.6	0.4	15.8
Abruption	1	0.1%	0	0.0	NA			
SROM	18	2.4%	1	0.5	0.990	0.2	0.0	1.6
SGA	10	1.4%	4	2.1	0.443	1.6	0.5	5.1

TSH, thyroid stimulating hormone; TGAb, anti-thyroglobulin antibodies; TPOAb, thyroperoxidase antibodies; SROM, spontaneous rupture of membranes; SGA, small for gestational age; FDIU, fetal death in utero.
